# Biological Effects of a Disposable, Canisterless Negative Pressure Wound Therapy System

**Published:** 2014-04-02

**Authors:** Malin Malmsjö, Elizabeth Huddleston, Robin Martin

**Affiliations:** ^a^Clinical Sciences, Lund, Lund University and Skåne University Hospital, Lund, Sweden; ^b^Advanced Wound Management Division, Smith and Nephew Medical Ltd, Hull, UK

**Keywords:** Blood flow, dressing, wound, NPWT, vacuum

## Abstract

**Objective:** Recent developments of negative pressure wound therapy (NPWT) systems have focused on making pumps smaller, lighter, and more portable. The recently introduced PICO system manages wound fluid through a highly breathable film within the dressing, thereby negating the need for a canister, which allows greater mobility and patient concordance. The aim of this study is to compare the biological effects of this system compared to a traditional NPWT system. **Methods:** Laboratory tests were carried out to demonstrate the fluid handling properties of the PICO™ system. Porcine full thickness defect wounds and sutured incisional wounds were used to compare the biological effects. Wounds were treated with PICO dressings or traditional NPWT dressings and connected to either a PICO device or a traditional NPWT device. **Results:** The PICO dressing manages exudate predominantly through evaporative loss (up to 85% of all fluid entering the dressing). Both traditional NPWT and the PICO system maintained therapeutic levels of negative pressure in all wounds. Both NPWT systems produced similar effects on wound edge contraction and microvascular blood flow in defect wounds. No significant changes in blood flow or wound contraction were noted in incision wounds for any NPWT combinations tested. **Conclusions:** The disposable, canisterless PICO NPWT system functions in the same manner as the traditional NPWT systems with regard to fluid handling, pressure transmission to the wound bed, tissue contraction, and changes in blood flow.

Negative pressure wound therapy (NPWT), in its modern form, was first described in 2 back to back articles in *Annals of Plastic Surgery* in 1997.^1^^,^^2^ The first article examined the mechanisms of action of NPWT in pigs^1^ and the second article described the clinical effects of NPWT on a wide range of acute, subacute, and chronic wounds.^2^ NPWT entails application of negative pressure to a sealed airtight wound. The wound is first filled with a wound filler (commonly gauze or foam) to allow pressure to be transmitted to and distributed evenly over the wound bed. The wound is then sealed with an adhesive film dressing and is connected to a vacuum pump via a drain or port. Wound fluid is drawn out and collected in a canister.

Current thoughts regarding the mechanisms of action of NPWT have largely been gained from animal and laboratory studies. Negative pressure wound therapy (NPWT) accelerates wound healing by initiating a cascade of interrelated biological effects in the wound edge that ultimately lead to wound healing. NPWT contracts the wound edges,^3^^,^^4^ stimulating angiogenesis^5^^,^^6^ and the formation of granulation tissue^7^ to expedite wound healing.

NPWT systems are evolving. Pumps are getting smaller, lighter, more portable, and nonreliant on mains or even battery power.^8^^-^11 Wound exudate canisters are becoming smaller. Dressing systems have been developed to make application and removal easier and less painful, offering a choice of wound fillers to tailor therapy to the needs of the wound and the patient.^12^^-^14 These developments allow greater utilization of a therapy that was once bulky, cumbersome, and expensive to use. Portability, discreetness, and simplicity of use may lead to greater patient well-being and compliance. However, as devices become smaller and more portable, manufacturers are faced with the challenge of ensuring that for equivalent wounds, small devices can be as effective as their larger counterparts.

The purpose of this study was to evaluate an ultraportable NPWT system that has been developed to provide NPWT in a simplified format. Exudate is managed predominantly by evaporation through the dressing, thereby negating the need for a canister, which makes the pump ultralight and portable. When a new system is introduced, it is crucial to understand and demonstrate whether such a system can deliver authentic NPWT and provide a therapeutic benefit. Preclinical tests for fluid handling, pressure transmission, wound edge contraction, and microvascular blood flow were studied to see whether the device functioned in the same way as the traditional, larger, and durable NPWT systems on both defect and closed incision wounds. These attributes have been widely employed as predictors of potential clinical performance.^1^^,^^4^^,^^12^^,^^15^

An emergent use of NPWT is its application as a postoperative dressing for closed surgical incisions.^16^ The majority of previous studies on the biological effects of NPWT have been carried out on defect wounds or open incision wounds where a wound-filler is used to fill a void prior to the application of negative pressure. It is not known whether the mechanisms of action arising from studies on defect wounds can be applied to all wound types, for example, where a defect is minimal or absent, such as, split thickness skin grafts, incisional wounds closed by sutures, staples or glue, or shallow wounds such as venous leg ulcers. This study was therefore performed using both a full-thickness defect wound model and a closed incision wound model in the pig.

## MATERIALS AND METHODS

### The NPWT systems used

**Figure 1:**
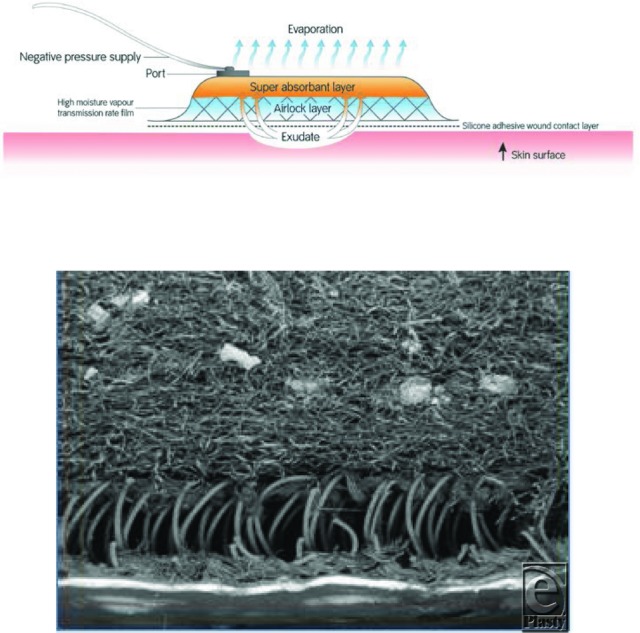
The structure and working principles of the PICO™ dressing. The PICO system simplifies NPWT by replacing the exudate canister with an absorbent dressing that has a high evaporative loss. The top panel illustrates wound exudate removal through the dressing. The PICO dressing is absorbent and is composed of 4 layers, as follows. The wound contact layer is a perforated flexible silicone adhesive layer, bonded to a lower airlock layer and an upper fluid absorption layer that delivers negative pressure, removes wound exudate, and aids evaporation of fluid through the high moisture vapor transmission rate upper film layer. The bottom panel is a scanning electron micrograph cross-section through a PICO dressing showing the 3 lower layers.

The PICO™ system consists of a single use pump (PICO device, Smith and Nephew Medical Ltd, Hull, UK), which delivers NPWT at a single preset pressure of −80 mm Hg and is disposable after 7 days of continual use. The device weighs 70 g, is powered by 2 AA lithium batteries with a single button control, and incorporates leak detection and low-battery indicators. The PICO dressing (Smith and Nephew Medical Ltd, Hull, UK) is composed of 4 layers (Fig 1), which serve to deliver NPWT and remove wound exudate predominantly through evaporative loss. The dressings have a wear time of up to 7 days and can be used either without fillers for shallow wounds or with traditional foam or gauze NPWT fillers for deeper cavities. For the purpose of these studies, the traditional NPWT system consisted of a RENASYS™ EZ device with foam or gauze dressings (RENASYS-Gauze or Foam Dressing kit and RENASYS EZ pump, Smith and Nephew, St Petersburg, Florida) set to deliver a continuous negative pressure of −80 mm Hg.

### Fluid handling

In vitro studies were carried out to quantitatively assess the fluid handling properties of the PICO system and concomitant delivery of NPWT. The 15 × 20 cm^2^ PICO dressings with or without a wound filler were applied over a 25-cm^2^ in vitro wound model. Simulated wound fluid (composed of a 1:1 dilution of horse serum: 0.9% saline) was delivered into the wound model at a rate of 1.1 mL/cm^2^/24 h, which is equivalent to levels of exudate typically removed by NPWT devices.^17^ The model was heated to 32°C to simulate skin temperature, and an air leak was introduced to reflect likely clinical applications, at a rate of 10 mL/min, which was determined as a worst case scenario based on recent clinical studies.^18^ Pressure in the base of the wound model was monitored continually over the test period. After 72 hours of PICO therapy, weight measurements were taken to establish how much fluid was retained in the dressing and how much fluid was lost to transpiration.

Further tests were performed to study the dynamics of fluid management over time at lower and upper extremes of flow rates. Low exudate rate was defined as 0.3 mL/cm^2^/24 h (a total of 7.5 mL/d) and high exudate rate was defined as 2.8 mL/cm^2^/24 h (a total of 70 mL/d). The wound model system was placed on a balance and the change in weight of the dressing was monitored continuously over the study period as fluid was introduced into the model. Wound bed pressure was monitored continually.

### In vivo wound model

An experimental in vivo wound model was used to test some of the recognized mechanical and biological properties of a NPWT system, including pressure distribution to the wound bed, tissue contraction, and altered patterns of blood flow.^4^^,^^15^ Studies were performed using a porcine peripheral wound model. The porcine study was approved by the Ethics Committee for Animal Research, Lund University, Sweden, which conforms to the principles outlined in the Declaration of Helsinki. All animals received humane care in compliance with the Guide for the Use and Care for Laboratory Animals as promulgated by the council of the American Physiologic Society and published by the National Institutes of Health, 1985. Details of anaesthesia and animal care were as previously described.^4^ Eight domestic Landrace pigs with a mean body weight of 70 kg were fasted overnight with free access to water. Animals were laid on their side and circular full-thickness wounds were created on the paravertebral area of the back, penetrating into the muscle. Defect wounds were made 6 cm in diameter and 2-cm deep. Incision wounds were made 10-cm long and 2-cm deep and then closed by suturing to model the prophylactic use of NPWT on closed incisions. Wounds were kept moist with saline. Temperature was monitored and maintained at 37°C. After the experiments, the animals were euthanized by injection of a lethal dose of potassium.

### Application of NPWT

In defect wounds, the wound cavity was either filled with polyurethane foam or gauze (RENASYS™-Gauze or Foam Dressing kit, Smith and Nephew, St Petersburg, Florida) or left empty. The sutured incision wounds were either covered with a small piece of foam or gauze or left bare. Both wound types were then covered with either a 15 × 20 cm^2^ PICO™ dressing, or a standard NPWT adhesive drape and wound drain combination (Chariker-Jeter method as previously described^4^). The NPWT dressings were connected either directly to a PICO prototype pump or a traditional NPWT pump (see earlier) set to deliver a negative pressure of −80 mm Hg. See Figure 3 for photos of the wounds.

### Wound pressure

To measure wound pressure following application of NPWT, a saline-filled pressure catheter was either sutured to the wound bed of defect wounds or on top of the suture line of incisional wounds. The pressure catheter was connected to a custom built pressure gauge as previously described.^4^

### Wound edge contraction

Wound contraction was measured as previously described.^4^ For defect wounds, 4 ink marks were made around the edge of the wound. The vertical and horizontal diameters of the wound were measured before and after the application of negative pressure, using a digital calliper. The mean of the diameters were calculated. For incisional wounds, 4 ink marks were made perpendicular and central to the incision line at a defined distance from the wound edge. Change in distance between opposite marks was measured before and after NPWT application.

### Wound edge microvascular blood flow

Microvascular blood flow was measured during therapy using Laser Doppler velocimetry, with a multichannel PeriFlux System 5000 (Perimed, Sweden) using 0.5-mm filament probes (Probe 418-1, Perimed) as previously described.^15^ A Venflon infusion cannula was used to gain access to the tissue. The filament probe was inserted through the cannula and the cannula was then removed, leaving the filament probe in place. In defect wounds, probes were placed into muscle tissue at distances of 0.5 cm and 2.5 cm from the wound edge. In incision wounds, probes were placed at the edge of the incision line at a depth of 0.5 cm and 2.5 cm from the skin surface. The filament probes were then connected to the laser Doppler equipment. Negative pressure dressing combinations were applied and blood flow was recorded after stabilization, which typically occurred within 2 minutes of NPWT application.

### Calculations and statistics

The order of application for the different treatment modalities was randomized in each wound. Calculations were performed using GraphPad 5.0 software (San Diego, California). Statistical analysis was performed using the Mann-Whitney test, when comparing 2 groups, and the Kruskal-Wallis test with Dunn's posttest for multiple comparisons, when comparing 3 groups or more. Significance was defined as *P* < 0.05. All differences referred to in the text were statistically significant. Results from the in vivo studies are presented as the mean of 8 measurements ± the standard error of the mean.

## RESULTS

### Fluid handling properties of the PICO system

Over a 72-hour period, an average of 81.5 mL of simulated wound exudate was delivered into the in vitro wound model, equating to a rate of 1.1 mL/cm^2^/24 h (Table 1). The tests demonstrate that on average, 80% of the fluid that had entered the dressing over the 72-hour period under negative pressure had evaporated, because only 20% of the fluid (13.8 mL) remained within the absorbant layer of the PICO dressing. The presence of a wound filler did not influence the fluid management properties of the PICO dressing, although it does retain some fluid itself (16%) due to its absorbent properties. In the absence of a wound filler, all of the fluid (100%) was removed from the wound directly by the dressing. Wound bed negative pressure was maintained around the set pressure of −80 mm Hg with a mean of −78.6 ± 2.6 mm Hg recorded at the bottom of the wound.

**Figure 2:**
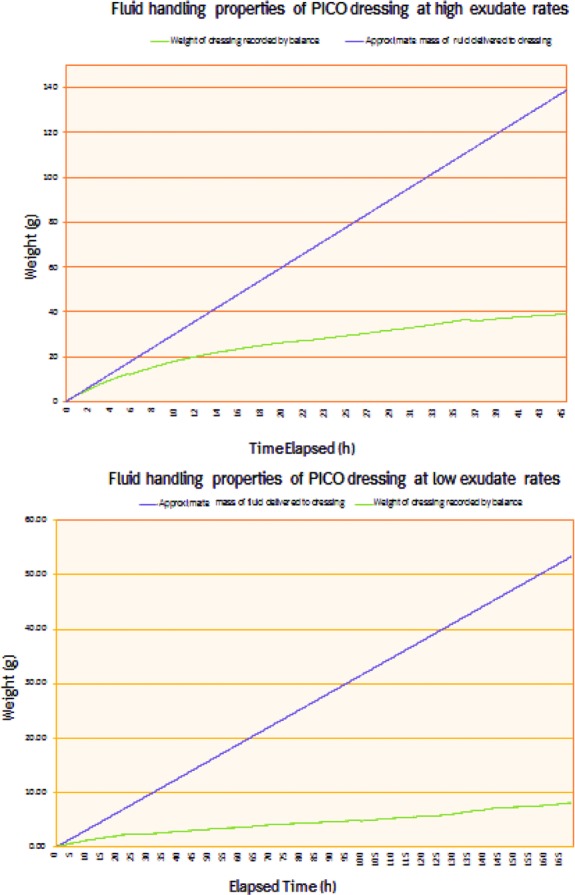
Fluid handling properties of the PICO™ system. Fluid was pumped into an in vitro wound model covered with a PICO dressing at low exudate rate of 7.5 mL/24 h for 7 days (*bottom* panel) and high exudate rate 70 of mL/24 h for 45 hours (*top* panel). Continuous weight measurements were taken to establish how much fluid was retained in the dressing and how much fluid was lost to transpiration. A background air leak at a rate of 10 mL/min was introduced to reflect possible clinical conditions of use. Results are shown as means values of 3 experiments.

**Figure 3:**
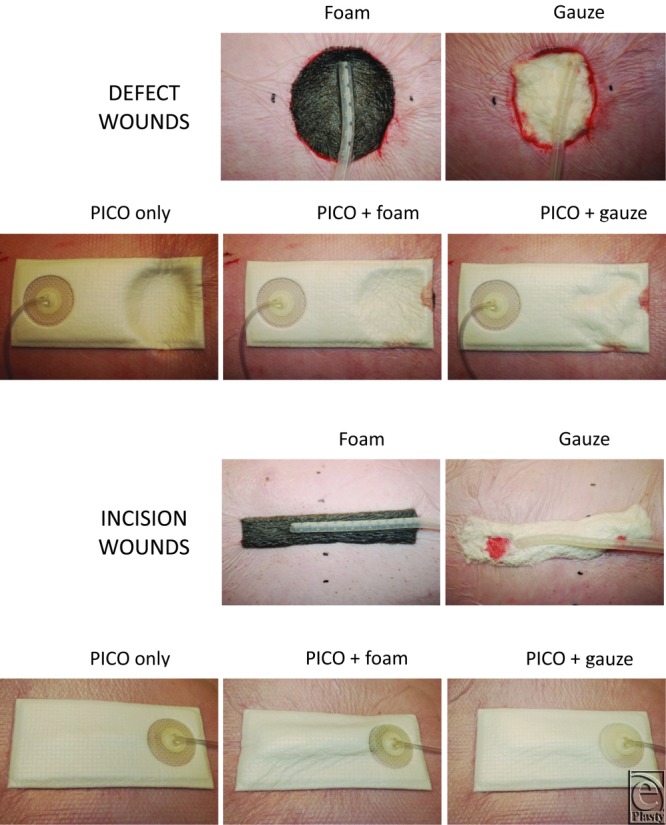
Photos of the treated wounds. Both incision and defect wounds were studied. In defect wounds, the wound cavity was either filled with polyurethane foam or gauze or left empty. The sutured incision wounds were either covered with a small piece of foam or gauze or left bare. Both wound types were then covered with either a 15 × 20 cm^2^ PICO™ dressing or a standard NPWT adhesive drape and wound drain. The NPWT dressings were connected either directly to a PICO prototype pump or a traditional NPWT pump set to deliver a negative pressure of −80 mm Hg. See “Methods” for details.

Continuous monitoring of fluid handling (Fig 2) confirms that a large proportion of fluid is removed from the dressing through evaporative loss irrespective of wound exudate rate. The proportion of fluid that is lost to evaporation increases with time. At high exudate rate (70 mL/d) up to 74% of the fluid entering the dressing was lost through evaporation over the study period. At low exudate levels (7.5 mL/d) up to 85% of the fluid was managed by evaporative loss through the dressing with up to 15% remaining in the super-absorber layer at any one time. Mean wound bed pressure was −79.1 ± 6.9 mm Hg at high exudate levels and −78.2 ± 6.6 mm Hg at low exudate levels.

### In vivo NPWT delivery

**Figure 4:**
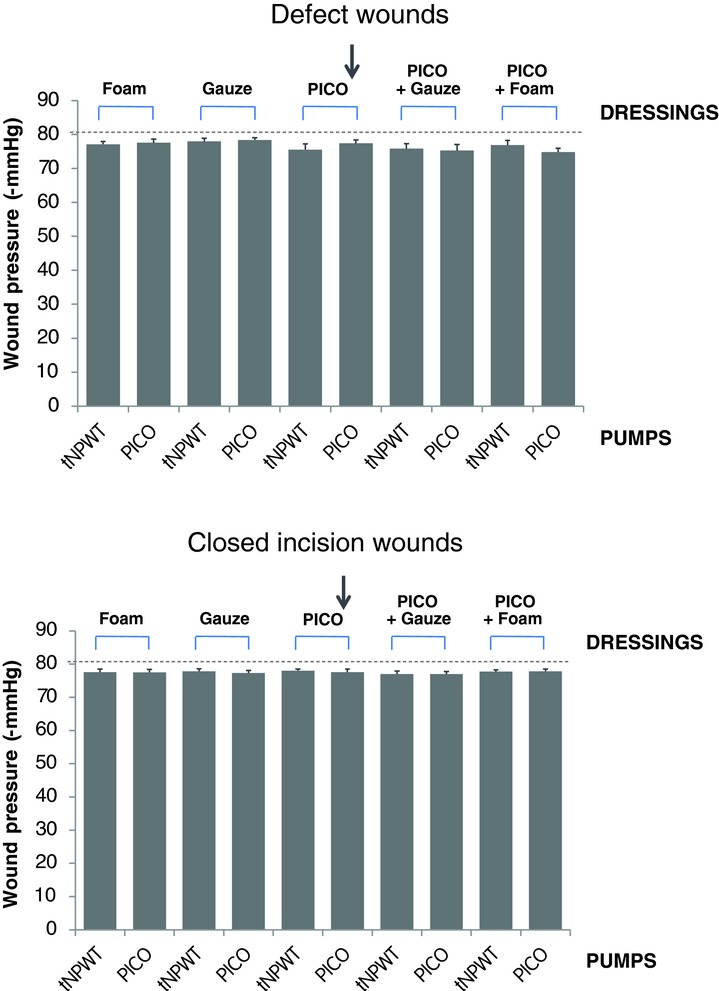
Pressure distribution, studied in vivo. Wounds were treated with different device, dressing and filler combinations set to provide NPWT at −80 mm Hg. The horizontal line (—) indicates the −80 mm Hg set point of the NPWT pumps. The arrow indicates the combination that represents the PICO™ system with no filler. The pressure was measured in the wound bed of defect wounds (*top* panel) or on top of the suture line of incisional wounds (*bottom* panel). Results are shown as means ± SEM of 8 experiments.

The PICO device and dressing were tested together and individually for the ability to deliver NPWT to the wound bed compared to traditional NPWT (Fig 4). In a defect wound, pressure distribution to the wound bed was similar for the PICO system (−77.4 ± 1.04 mm Hg) and the traditional NPWT system (−77.6 ± 0.86 mm Hg (*P* = nonsignificant [ns]), regardless of wound filler. In closed incision wounds, where the probe was positioned over the suture line, the pressure recorded across all wounds was similar, irrespective of dressing and device combination, with a mean recording of −77.6 ± 0.23 mm Hg (*P* = ns).

### In vivo wound contraction

**Figure 5:**
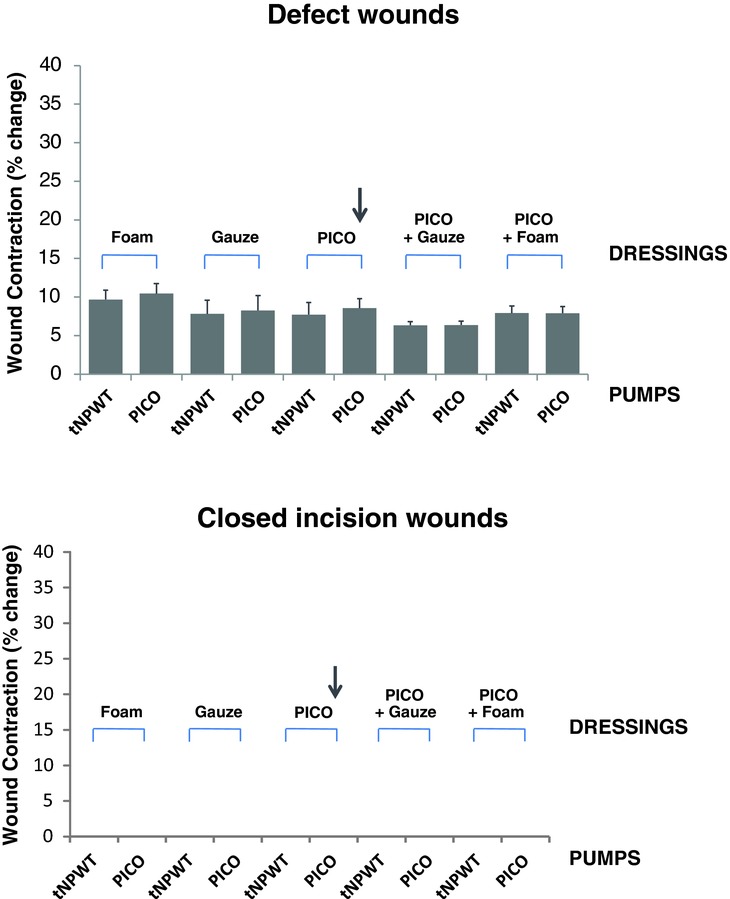
Wound contraction. Wounds were treated with different device, dressing, and filler combinations set to provide NPWT at −80 mmHg. For defect wounds, the vertical and horizontal diameters of the wound, and for incisional wounds, distance between previously defined landmarks from the wound edge, were measured before and after the application of negative pressure and the mean value of the percent change was calculated. The arrow indicates the combination that represents the PICO™ system with no filler. Results are shown as means ± SEM of 8 experiments.

In defect wounds, the PICO system and the traditional NPWT system stimulate similar wound contraction (between 6% and 10% reduction in original wound area). Contraction was slightly, but not significantly, greater when the wound was filled with foam than with gauze (*P* = ns). In contrast, no contraction was observed in incision wounds regardless of NPWT treatment modality (Fig 5).

### In vivo wound edge microvascular blood flow

**Figure 6:**
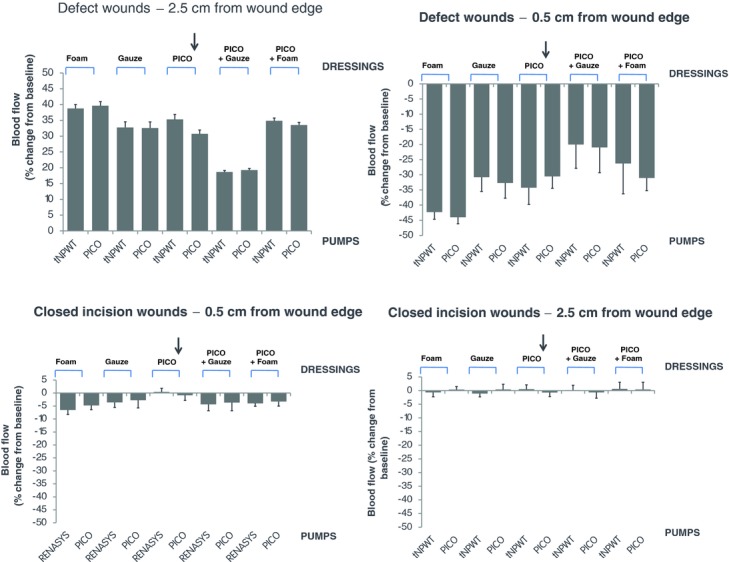
Wound edge microvascular blood flow. Wounds were treated with different device, dressing, and filler combinations set to provide NPWT at −80 mm Hg. Microvascular blood flow in the wound edge was measured using laser Doppler velocimetry before and after application of NPWT. In defect wounds, the probes were placed 0.5 cm and 2.5 cm from the wound edge. In incision wounds, the probes were placed at a depth of 0.5 cm and 2.5 cm from the skin surface, close to the incision edge. Microvascular blood flow is expressed as percentage change relative to baseline values. The arrow indicates the combination that represents the PICO™ system with no filler. Results are shown as means ± SEM of 8 experiments.

In defect wounds, the PICO system and the traditional NPWT system create similar microvascular blood flow changes (Fig 6, *P* = ns). At 2.5 cm from the wound edge, there was an increase in blood flow (between 20% and 40%), and closer to the wound edge (0.5 cm), there was a decrease in blood flow (between −40% and −20%). The blood flow changes appeared to be greater when the wound was filled with foam than when the wound was filled with gauze; however, this did not reach statistical significance (*P* = ns).

On the whole, there is little effect on hyperperfusion or hypoperfusion in incisional wounds for any of the NPWT system combinations tested (Fig 6). However, a slight decrease in blood flow was observed in the superficial tissue (0.5-cm deep) in those wounds where wound fillers were placed over the incision line.

## DISCUSSION

Recent developments of NPWT systems have focused on making pumps smaller and lighter to facilitate portability, discreetness, and simplicity of use and to assist patient well-being and concordance. The recently introduced PICO system manages wound fluid through a highly breathable film within the dressing which removes the need for a canister and makes the pump ultralight and portable.

### Fluid handling

The results from this study show that the PICO™ system handled clinically relevant volumes of fluid at both high and low exudate conditions and demonstrate how wound fluid is removed by the PICO dressing through absorption and subsequent evaporation through a high-moisture vapor transmission rate upper film. These studies were carried out under carefully controlled conditions; however, in reality, the rate of evaporative loss of fluid through the dressing (and therefore the life of the dressing) will of course depend on many different factors, such as, temperature and humidity of the surrounding environment, wear time, exudate rates, and choice of dressing size. In practical terms, these in vitro tests document the ability of the NPWT dressing to handle fluid from a typical 25 cm^2^ wound that might in the course of a week fill a traditional NPWT device 300 mL canister with almost 200 mL of wound exudate. When the PICO system is used with foam or gauze, a mid-week dressing replacement would be needed in accordance with the instructions for use of foam or gauze NPWT dressings. In less exuding wounds, not treated with foam or gauze, the PICO dressing may remain in place for the 7-day lifetime of the PICO pump, as shown in a clinical study of the PICO system.^18^

### Pressure distribution and wound contraction

The biological effects of NPWT that ultimately lead to wound healing rely entirely on transduction of negative pressure to the wound bed and tissue edges. This is usually mediated by foam or gauze wound fillers to ensure delivery of therapeutic levels of pressure. The PICO system was equally effective in delivering negative pressure to the wound bed and causing wound contraction as the traditional NPWT system. It is well known that the pressure is only distributed to the wound tissue that is in immediate contact with the wound dressing or filler. Interestingly, this work revealed that the dressing effectively communicates negative pressure to the wound bed without the need for fillers. In defect wounds, pressure is transmitted through the wound filler and dressing to the bottom of the wound and the resulting pressure differential will result in drainage of fluid from the entire wound cavity and contraction of the wound edges. In closed incision wounds, the suture line is exposed to the negative pressure, but it is not understood if it reaches any of the deeper wound structures. Understandably, no changes in contraction are expected in closed incisional wounds because there is no dead space or void to contract. Indeed, it is thought that the NPWT-related biomechanical forces applied to incision wounds serve to bolster the wound and reduce the points of tension that can be created by closure methods such as sutures and staples.^19^

### Microvascular blood flow

The PICO system operates in an identical fashion to traditional NPWT systems in setting up the same patterns of blood flow. In defect wounds, there was a decrease in blood flow close to the wound edge (0.5 cm) and an increase in blood flow at 2.5 cm from the wound edge, which has been shown in numerous studies earlier.^20^^,^^21^ An interesting relationship between blood flow changes and extent of tissue contraction is apparent in these studies. Where contraction was noted to be greatest, blood flow changes seemed also to have the largest changes (both hyperperfusion and hypoperfusion). This was also consistent with the pattern on incisional wounds where all NPWT combinations created negligible wound contraction and little effects on blood flow were observed.

The decrease in microvascular blood flow observed during NPWT application may occur from the resultant forces of the tissue being pulled toward the suction force and compressing against the wound filler or dressing, thus constricting the vasculature.^22^ The increase in blood flow is harder to explain. When NPWT is applied to a defect wound, the pulling effect may stretch the deeper tissues and open up vascular beds. This proposed link between contraction and blood flow is further supported by the observation in this study that blood flow effects were slightly greater for wounds filled with foam than wounds filled with gauze. This may be because foam results in slightly greater wound contraction than gauze due to its more compressive nature.

In incision wounds, NPWT resulted in a slight decrease in blood flow in the superficial tissue (0.5-cm deep) when a wound filler was placed over the incision line. This hypoperfusion may again be explained by the compressive forces of the wound filler against the tissue.

Both the increase and decrease in blood flow may be beneficial for the wound. Hyperperfusion may ensure adequate oxygenation and nutrient supply and removal of waste products from the healing wound.^23^ Hypoperfusion may stimulate angiogenesis and granulation tissue formation, which in turn facilitate the process of wound healing.^2^^,^^24^ However, in tissues with impaired circulation, this may not be a desirable outcome, and it has been suggested that NPWT should be applied with caution to tissues with compromised vascularity.^25^^-^27

### Biological effects of NPWT in different wounds types

This study demonstrates that not all wounds are the same, and NPWT may work though a different mix of the multiple mechanisms of action in different wound types, depending on the geometry of the wound. More studies are needed to increase our understanding of the mode of action of NPWT in other wounds such as incisional or shallow wounds where there is little component of tissue contraction. Applying NPWT to closed incisions has been demonstrated to offer a number of clinical benefits including earlier cessation of wound drainage, reduction in seromas and hematomas, and a reduction in dehiscence and infection rates in high-risk patients.^16^^,^^28^^-^31 Despite this, little is known about the main mechanisms of action of NPWT in this indication. Animal studies propose that NPWT may accelerate clearance of hematoma and seromas through the lymphatic system^32^ and improve the distribution of lateral tension over the incision.^19^ A recent systematic review of the evidence for NPWT on closed incisions discusses potential mechanisms of action.^33^

## CONCLUSION

The means by which the PICO™ NPWT system manages exudate is fundamentally different from the majority of NPWT systems, which traditionally use a fluid canister. The present studies show that fluid is effectively managed by the PICO dressing; approximately 80% is evaporated through the film component of the dressing, with the remaining fluid held above the wound surface in the absorbent components. Nevertheless, the PICO system maintains the ability to deliver the key functional properties of NPWT systems: communication of negative pressure to the wound bed, wound edge tissue contraction, and changes in patterns of microvascular blood flow. NPWT may have different roles to play in different wound types and this warrants further investigation.

## Figures and Tables

**Table 1:** Fluid handling properties of the PICO™ system*.

		Amount of fluid			
	Total fluid delivered to the wound	Delivered to PICO dressing	Remaining in wound filler	Remaining in dressing after 72 h	Lost through evaporation
With gauze wound filler	79.0 mL ±2.0	66.0 mL (84%) ±1.0	13.0 mL (16%) ±1.0	13.0 mL (20%) ±1.0	53.0 mL (80%) ±1.0
Without wound filler	82.0 mL ±2.0	82.0 mL (100%) ±2.0	—	14.5 mL (18%) ±0.5	67.5 mL (82%) ±2.5

*Simulated exudate was introduced into an in vitro wound model at a flow rate of 1.1 mL/cm^2^ wound area per 24 hours. The 25-cm^2^ wounds were covered with a 15 × 20 cm^2^ PICO dressing with or without gauze wound filler. Weight measurements were taken after 72 hours of NPWT to establish how much fluid was retained in the dressing or filler or lost to transpiration (n = 2, mean ± SEM; 1 g of weight was assumed equivalent to 1 mL fluid).

## References

[1] Morykwas MJ, Argenta LC, Shelton-Brown EI, McGuirt W (1997). Vacuum-assisted closure: a new method for wound control and treatment: animal studies and basic foundation. Ann Plast Surg.

[2] Argenta LC, Morykwas MJ (1997). Vacuum-assisted closure: a new method for wound control and treatment: clinical experience. Ann Plast Surg.

[3] Borgquist O, Gustafsson L, Ingemansson R, Malmsjo M (2010). Micro- and macromechanical effects on the wound bed of negative pressure wound therapy using gauze and foam. Ann Plast Surg.

[4] Malmsjo M, Ingemansson R, Martin R, Huddleston E (2009). Negative-pressure wound therapy using gauze or open-cell polyurethane foam: similar early effects on pressure transduction and tissue contraction in an experimental porcine wound model. Wound Repair Regen.

[5] Chen SZ, Li J, Li XY, Xu LS (2005). Effects of vacuum-assisted closure on wound microcirculation: an experimental study. Asian J Surg.

[6] Greene AK, Puder M, Roy R (2006). Microdeformational wound therapy: effects on angiogenesis and matrix metalloproteinases in chronic wounds of 3 debilitated patients. Ann Plast Surg.

[7] Borgquist O, Gustafsson L, Ingemansson R, Malmsjö M (2010). Micro- and macromechanical effects on the wound bed of negative pressure wound therapy using gauze and foam. Ann Plast Surg.

[8] Sposato G, Molea G, Di Caprio G, Scioli M, La Rusca I, Ziccardi P (2001). Ambulant vacuum-assisted closure of skin-graft dressing in the lower limbs using a portable mini-VAC device. Br J Plast Surg.

[9] Bendewald FP, Cima RR, Metcalf DR, Hassan I (2007). Using negative pressure wound therapy following surgery for complex pilonidal disease: a case series. Ostomy Wound Manage.

[10] Landsman A (2010). Analysis of the SNaP Wound Care System: a negative pressure wound device for treatment of diabetic lower extremity wounds. J Diabetes Sci Technol.

[11] Khanbhai M, Fosah R, Oddy MJ, Richards T (2012). Disposable NPWT device to facilitate early patient discharge following complex DFU. J Wound Care.

[12] Malmsjo M, Gustafsson L, Lindstedt S, Gesslein B, Ingemansson R (2012). The effects of variable, intermittent, and continuous negative pressure wound therapy, using foam or gauze, on wound contraction, granulation tissue formation, and ingrowth into the wound filler. Eplasty.

[13] Fraccalvieri M, Zingarelli E, Ruka E (2011). Negative pressure wound therapy using gauze and foam: histological, immunohistochemical and ultrasonography morphological analysis of the granulation tissue and scar tissue. Preliminary report of a clinical study. Int Wound J.

[14] Dorafshar AH, Mieczyslawa F, Lohman R, Gottlieb LJ (2009). Prospective randomized study comparing gauze suction negative pressure wound therapy with standard vacuum assisted closure device. Paper presented at: the 88th Annual Meeting and Symposium of American Association of Plastic Surgeons.

[15] Malmsjo M, Ingemansson R, Martin R, Huddleston E (2009). Wound edge microvascular blood flow: effects of negative pressure wound therapy using gauze or polyurethane foam. Ann Plast Surg.

[16] Stannard JP, Volgas DA, McGwin G (2012). Incisional negative pressure wound therapy after high-risk lower extremity fractures. J Orthop Trauma.

[17] Dealey C, Cameron J, Arrowsmith M (2006). A study comparing two objective methods of quantifying the production of wound exudate. J Wound Care.

[18] Hudson DA, Adams KG, Huyssteen AV, Martin R, Huddleston EM Simplified negative pressure wound therapy: clinical evaluation of an ultraportable, no-canister system [published online ahead of print May 7, 2013]. Int Wound J.

[19] Wilkes RP, Kilpad DV, Zhao Y, Kazala R, McNulty A (2012). Closed incision management with negative pressure wound therapy (CIM): biomechanics. Surg Innov.

[20] Borgquist O, Anesater E, Hedstrom E, Lee CK, Ingemansson R, Malmsjo M (2011). Measurements of wound edge microvascular blood flow during negative pressure wound therapy using thermodiffusion and transcutaneous and invasive laser Doppler velocimetry. Wound Repair Regen.

[21] Borgquist O, Ingemansson R, Malmsjo M (2010). Wound edge microvascular blood flow during negative-pressure wound therapy: examining the effects of pressures from −10 to −175 mm Hg. Plast Reconstr Surg.

[22] Kairinos N, Solomons M, Hudson DA (2010). The paradox of negative pressure wound therapy: in vitro studies. J Plast Reconstr Aesthet Surg.

[23] Jonsson K, Jensen JA, Goodson WHI (1991). Tissue oxygenation, anemia, and perfusion in relation to wound healing in surgical patients. Ann Surg.

[24] Petzina R, Gustafsson L, Mokhtari A, Ingemansson R, Malmsjo M (2006). Effect of vacuum-assisted closure on blood flow in the peristernal thoracic wall after internal mammary artery harvesting. Eur J Cardiothorac Surg.

[25] Kairinos N, Voogd AM, Botha PH (2009). Negative-pressure wound therapy II: negative-pressure wound therapy and increased perfusion. Just an illusion?. Plast Reconstr Surg.

[26] Venturi ML, Attinger CE, Mesbahi AN, Hess CL, Graw KS (2005). Mechanisms and clinical applications of the vacuum-assisted closure (VAC) Device: a review. Am J Clin Dermatol.

[27] Attinger CE, Janis JE, Steinberg J, Schwartz J, Al-Attar A, Couch K (2006). Clinical approach to wounds: debridement and wound bed preparation including the use of dressings and wound-healing adjuvants. Plast Reconstr Surg.

[28] Stannard JP, Robinson JT, Anderson ER, McGwin G, Volgas DA, Alonso JE (2006). Negative pressure wound therapy to treat hematomas and surgical incisions following high-energy trauma. J Trauma.

[29] Reddix RN, Tyler HK, Kulp B, Webb LX (2009). Incisional vacuum-assisted wound closure in morbidly obese patients undergoing acetabular fracture surgery. Am J Orthop (Belle Mead NJ).

[30] Atkins BZ, Wooten MK, Kistler J, Hurley K, Hughes GC, Wolfe WG (2009). Does negative pressure wound therapy have a role in preventing poststernotomy wound complications?. Surg Innov.

[31] Gomoll AH, Lin A, Harris MB (2006). Incisional vacuum-assisted closure therapy. J Orthop Trauma.

[32] Kilpadi DV, Cunningham MR (2011). Evaluation of closed incision management with negative pressure wound therapy (CIM): hematoma/seroma and involvement of the lymphatic system. Wound Repair Regen.

[33] Karlakki S, Brem M, Giannini S, Khanduja V, Stannard J, Martin R (2013). Negative pressure wound therapy for managementof the surgical incision in orthopaedic surgery: A review of evidence and mechanisms for an emerging indication. Bone Joint Res.

